# Effect of quercetin on the number of blastomeres, zona pellucida thickness, and hatching rate of mouse embryos exposed to actinomycin D: An experimental study

**Published:** 2018-02

**Authors:** Hamid Reza Sameni, Sara Sadat Javadinia, Manouchehr Safari, Mohammad Hasan Tabrizi Amjad, Nasrin Khanmohammadi, Houman Parsaie, Sam Zarbakhsh

**Affiliations:** *Research Center of Nervous System Stem Cells, Department of Anatomy, Faculty of Medicine, Semnan University of Medical Sciences, Semnan, Iran* **.**

**Keywords:** Quercetin, Embryonic development, Zona pellucida, Apoptosis, Blastocyst inner cell mass

## Abstract

**Background:**

Quercetin is a flavonoid with the ability to improve the growth of embryos in vitro, and actinomycin D is an inducer of apoptosis in embryonic cells.

**Objective:**

The aim was to evaluate the effect of quercetin on the number of viable and apoptotic cells, the zona pellucida (ZP) thickness and the hatching rate of preimplantation embryos exposed to actinomycin D in mice.

**Materials and Methods:**

Two-cell embryos were randomly divided into four groups (Control, Quercetin, actinomycin D, and Quercetin + actinomycin D group). Blastocysts percentage, hatched blastocysts, and ZP thickness of blastocysts was measured. The number of blastomeres was counted by Hoechst and propidium iodide staining and the apoptotic cells number was counted by TUNEL assay.

**Results:**

The results showed that the use of quercetin significantly improved the growth of embryos compared to the control group (p=0.037). Moreover, quercetin reduced the destructive effects of actinomycin D on the growth of embryos significantly (p=0.026).

**Conclusion:**

quercetin may protect the embryos against actinomycin D so that increases the number of viable cells and decreases the number of apoptotic cells, which can help the expansion of the blastocysts, thinning of the ZP thickness and increasing the hatching rate in mouse embryos.

## Introduction

In vitro production of the embryo is an important method for improving reproductive technology. The development potential of embryos produced by in vitro production is related to the composition of the culture medium (1). It is known that embryo development in culture media can be damaged by several stressors such as high oxygen concentration and high level of reactive oxygen species (ROS) (2). It is important to keep embryos in culture media from oxidative stress and for this purpose, antioxidants are valuable candidates (3).

Flavonoids are phyto phenolic compounds with the strong antioxidant effect that work as free radical scavengers. Quercetin is an important member of flavonoid family in the human diet which is abundant in fruits and vegetables such as, apple, onion, strawberry, tea, green tea and broccoli (4, 5). It has been reported that quercetin has anti-oxidative, anti-inflammatory and anti-mutagenic activities due to free radical scavenging so that quercetin reduces anomalies in rat embryos, decreases neural tube defects in embryos of diabetic mice and reduces hair cell damage in zebrafish embryos (5-8).

Actinomycin D is a DNA ligand drug that clinically used as an antibiotic to treat cancer. Actinomycin D inhibits DNA transcription by connecting to guanine-cytosine base pairs (9). In some reports, actinomycin D has been used to induce apoptosis in preimplantation embryos (10, 11). By studying the embryonic morphology, predicting the fate of the embryos is largely possible. The most morphological indicators to select the best embryos for transfer are zona pellucida (ZP) thickness and the number of embryonic cells (12). ZP thickness is a reliable indicator of in vitro fertilization success rate that can be applied as a criterion for embryo selection. Actually, ZP thickness is inversely correlated with embryonic viability and hatching rate (13, 14). 

Moreover, cleavage rate and development to blastocyst are applied as a quality parameter of mammal embryos (12, 15). In the previous study, we showed l-carnitine as an antioxidant could increase embryo development and blastocyst quality but it is still unclear which antioxidant is more effective in embryo culture and so different antioxidants should be investigated (16). Recent studies have reported quercetin has the protective effect on the development of mouse embryos against H_2_O_2_ and hydroxyurea (17, 18).

The aim of this study was to evaluate the antioxidant and anti-apoptotic effects of quercetin on some morphological indicators of preimplantation embryos in vitro. The morphological indicators include the ZP thickness, the number of viable and apoptotic blastomeres and the hatching rate. To evaluate the antioxidant and anti-apoptotic properties of quercetin, actinomycin D was used as an inducer of apoptosis in blastomeres.

## Materials and methods

**Animals**

In this experimental study, female C57BL/6 mice (6-8 wk) were kept under controlled temperature (25±2^o^C) and controlled light (12 hr light/dark), with free access to food and water. 

**Superovulation and embryo collection**

The mice were superovulated by an intraperitoneal injection of 10 IU pregnant mare's serum gonadotropin (Gonaser 5.000 UI, Spain) followed 48 hr later by an intraperitoneal injection of 10 IU human chorionic gonadotropin (hCG) (Sigma, China) (19). Then they were mated overnight with males and the mating was emphasized by the presence of vaginal plug on the morning after hCG injection. Two-cell embryos were flushed from the oviduct at about 48 hr after hCG injection and washed in human tubal fluid (HTF) medium containing HEPES (Sigma, USA). From 20 female mice, a total of 240 two-cell embryos were obtained and used in this study.

**Embryo culture**

The 240 two-cell embryos were transferred into the HTF medium (supplemented with 10% human serum albumin) (Sigma, USA). Two-cell embryos were randomly divided into four groups (60 embryos in each group): I. Control group, without any treatment, II. Quercetin (Sigma, China) group, 5 µM of quercetin (17) was added into the medium, III. Actinomycin D (Sigma, USA) group, 0.005 µg/ml of actinomycin D (10) was added into the medium, IV. Quercetin + actinomycin D group, 5 µM of quercetin and 0.005 µg/ml of actinomycin D were added together into the medium. 

In all groups, 10 embryos were situated in a drop of 20 μl of HTF medium under mineral oil (Sigma, USA) in a 35 mm Petri dish (Jet Biofil, Canada) and were incubated at 37^o^C with 95% humidity and 5% CO_2_. To evaluate the anti-apoptotic effect of quercetin, when the two-cell embryos reached the eight-cell stage, they were incubated with 0.005 µg/ml of actinomycin D in the medium for 4 hr. Actinomycin D concentration was derived from the other report (10). In 120 hr after the onset of incubation, the percentage of embryos that reached to blastocyst and hatched blastocyst stages was assessed.

**Measurement of ZP thickness**

The ZP thickness measurement was performed in two stages of the early blastocyst and full blastocyst. First, blastocysts were randomly selected. Then the thickness of each ZP was measured at three steady points and the average was calculated (12, 16). The measurement was taken from images using an inverted microscope (Nikon, Eclipse Ti-U, Japan) and motic images plus 2.0 software.

**Differential staining and detection of apoptotic nuclei**

When the blastocysts were expanded, randomly selected for counting the number of total blastomeres by differential staining and TUNEL assay. Differential staining to separate the inner cell mass and trophectoderm cells and TUNEL assay to detect the apoptotic nuclei were done according to the method described by Fouladi-Nashta and colleagues (20, 21). Briefly, the blastocysts were treated with 30 μg/ml propidium iodide (PI) (Sigma, China) and 1% Triton X-100 (Sigma, China) at 37^o^C for 5 min. Immediately after, the blastocysts were washed twice and fixed in 4% paraformaldehyde containing 10 μg/ml bisbenzimide (Hoechst 33342) (Sigma, USA) for 20 min at room temperature that allows fixation of blastocysts and staining total cell nuclei. 

Next, the blastocysts were washed and incubated in droplets of in situ cell death detection (TUNEL) kit solution (Roche, Germany) for 45 min according to the manufacturer`s instructions. Then the blastocysts were mounted on glass slides in glycerol droplets and were observed under an inverted fluorescent microscope (Motic, AE31, Spain). Inner cell mass (ICM) nuclei labeled with Hoechst appeared blue, trophectoderm (TE) nuclei labeled with both Hoechst and PI appeared red and apoptotic cells labeled with TUNEL appeared green. The number of ICM, TE and apoptotic cells was counted.

**Ethical consideration**

All animal protocols were approved by the Research Council of of Semnan University of Medical Sciences (IR.SEMUMS.REC.1394. 83).

**Statistical analysis**

Comparison of the percentage of embryos from two-cell to hatched blastocyst was analyzed by x^2^ test. The ZP thickness, the blastocyst cell count and the TUNEL positive cell count were analyzed by independent t-test as mean ± standard deviation. A difference with p<0.05 was considered statistically significant.

## Results

**Developmental rate of embryos**

**Figure 1:**
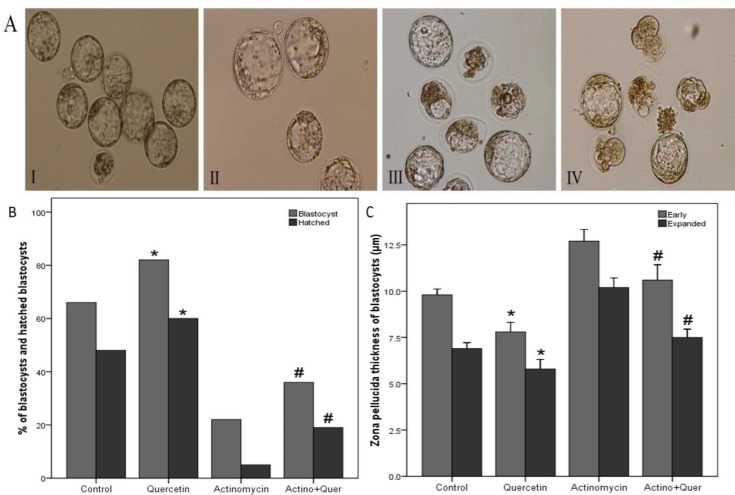
A. The blastocysts of I) Control, II) Quercetin, III) Actinomycin D, and IV) Quercetin + actinomycin D (×200). B. The results of the percentage of blastocysts and hatched blastocysts. C. The results of zona pellucida thickness of early and expanded blastocysts

There was no statistically significant difference in the percentage of two-cell embryos to morula stage between quercetin and control groups (p=0.559); and between quercetin + actinomycin D and actinomycin D groups (p=0.690) (Table I). The percentage of embryos that reached to blastocyst and hatched blastocyst stages in quercetin group was significantly higher than the control group (p=0.037), and in quercetin+ actinomycin D group was significantly higher than actinomycin D group (p=0.026) (Figure 1A, B). 

**The ZP thickness of blastocysts**

The results of ZP thickness of blastocysts showed the ZP thickness in quercetin group was significantly thinner than the control group (p=0.043), and in quercetin + actinomycin D group was significantly thinner than actinomycin D group (p=0.027) (Figure 1A, C). 

**Blastocyst cell count**

**Figure 2:**
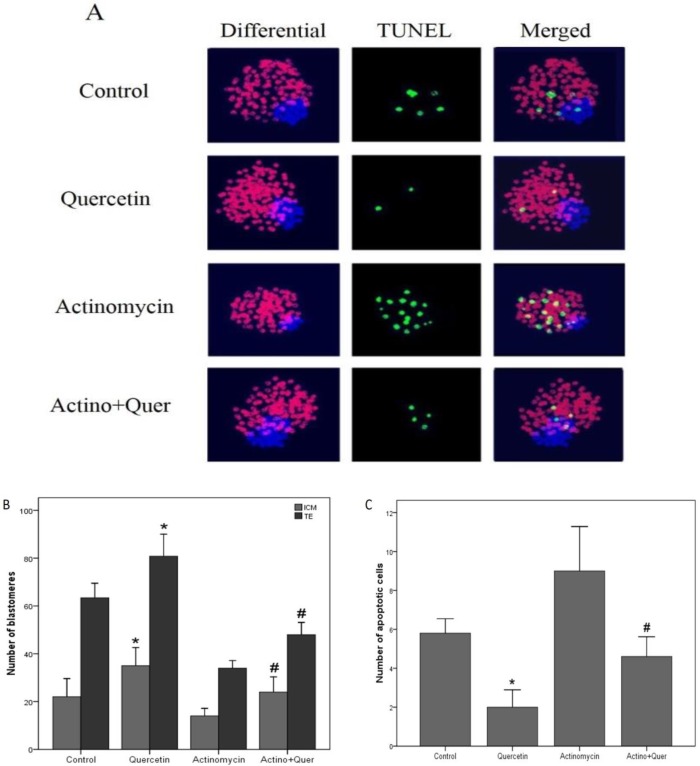
A. Differential staining and TUNEL labeling of blastocysts. The triple staining with propidium iodide (PI) for trophectoderm cells (red), Hoechst for total cells (blue), and TUNEL for apoptotic cells (green) (×200). B. The results of blastocyst cell numbers with PI and Hoechst staining. C. The results of apoptotic cells of blastocysts with TUNEL assay.

Expanded blastocysts were stained with Hoechst and PI and the number of ICM and TE was counted. The results showed the number of ICM and TE in quercetin group was significantly higher than the control group (p=0.034), and in quercetin+ actinomycin D group was significantly higher than actinomycin D group (p=0.037) (Figure 2A, B). 

**Anti-apoptotic effect of quercetin**

The TUNEL assay was used to determine the anti-apoptotic effect of quercetin on blastocyst development in the presence or absence of actinomycin D. The results showed the number of TUNEL-positive nuclei in quercetin group was significantly lower than control group (p=0.011), and in quercetin+ actinomycin D group was significantly lower than actinomycin D group (p=0.019) (Figure 2A, C). 

**Table I:** two-cell embryos up to morula stage in different groups

**Groups**	**2-Cell**	**4-Cell**	**8-Cell**	**Morula**
Control	60 (100)	60 (100)	59 (98.33)	58 (96.66)
Quercetin	60 (100)	60 (100)	60 (100)	59 (98.33)
Actinomycin D	60 (100)	59 (98.33)	58 (96.66)	41 (68.33)
Quercetin+Actinomycin D	60 (100)	60 (100)	58 (96.66)	43 (71.66)

Data presented as n (%)

*p<0.05 vs. the control group.

#p<0.05 vs. the actinomycin D group.

*p<0.05 vs. the control group.

#p<0.05 vs.the actinomycin D group.

## Discussion

In this study, we evaluated the effect of quercetin on some morphological indicators of mouse embryo development and blastocyst quality including the ZP thickness, the hatching rate, the number of blastomeres and the rate of apoptosis induced by actinomycin D. Overall, the results showed 5 µM of quercetin was able to promote the development of embryos exposed to actinomycin D and reduced apoptosis in the blastomeres, suggesting quercetin as an antioxidant had a protective effect on mouse embryo development and blastocyst quality against actinomycin D. 

Quercetin is one of the most important flavonoids. The antioxidant capacity of quercetin has been shown in different cell types (22, 23). In relation to the effect of quercetin on mouse embryo development, Yu *et al* have reported that quercetin has the protective effect on mouse embryos against H_2_O_2_ (17). In the other study, Perez-Pasten and co-worker have reported that using more than 100 µM of quercetin causes some defects and anomalies, and 3 µM of quercetin has the protective effect on mouse embryos against hydroxyurea (18). Moreover, Shahzad *et al* have reported that administration of 50 mg/kg/day to pregnant rats decreased implantation rate (24). It seems that high doses of antioxidants may act as enzyme inhibitors and mutagens due to inhibition of topoisomerases, proteasome synthesis or fatty-acid synthesis (25, 26). To induce apoptosis, we used actinomycin D which is known as an inducer of apoptosis on different cell types by inhibiting DNA transcription (11, 27). Some articles have shown that actinomycin D decreases development and cleavage rate of embryos, and increases apoptotic cells in blastocyst (10, 11, 28).

The results showed the quercetin improved development of two-cell embryos to hatched blastocyst stage and reduced apoptotic cells of the embryos exposed to actinomycin D. These results are in accordance with the other reports that have shown quercetin has protective effects on mouse embryos against H_2_O_2_ and hydroxyurea (17, 18). Due to the different mechanism of actinomycin D with H_2_O_2_ and hydroxyurea, our results confirmed quercetin could protect mouse embryos in vitro from various harmful factors. These effects of quercetin are probably related to its antioxidant action in reducing ROS level because it is well known that ROS induces apoptotic cell death in different cell types (29, 30). Moreover, quercetin protects the mitochondrial function (31) and regulates enzyme antioxidant defense systems (32). In the other study, Sovernigo *et al* have reported that quercetin can improve bovine blastocyst development (33). The results of our study are in agreement with Fan and Sovernigo reports.

By studying morphological indicators, embryo quality is checked and the best embryos are selected for transfer (34-36). From the most important indicators of embryonic morphology are the number of viable and apoptotic cells of the blastocyst, the ZP thickness of blastocyst and the ability of hatching (37, 38). The number of blastocyst cells is important for appropriate implantation and various studies have reported that reduction in the number of blastocyst cells leads to reduced embryonic viability (39, 40). Blastocoel fluid has H_2_O_2_ that is cytotoxic and induces apoptosis in blastocyst cells (41). Antioxidant effect of quercetin probably increases intracellular glutathione level and because glutathione is involved in the removal of H_2_O_2_, blastocyst cells would not become apoptosis (6). Our results showed quercetin increased the number of viable cells of the blastocyst and decreased apoptotic cells that these results were in agreement with the other report (17).

ZP thickness is a marker to select the best frozen-thawed embryos for transfer (42) because the thinner ZP increases the probability of the hatching rate and implantation. ZP thickness depends on inherent features of embryos to generate the lytic factors needed for ZP thinning (13, 43). Khanmohammadi and colleagues showed l-carnitine could cause thinning of the ZP thickness of blastocysts (16). Prior to the present study, the effect of quercetin on ZP thickness has not been investigated. We showed that quercetin could cause thinning of the ZP thickness of blastocysts. It seems that blastocyst expansion depends on the number of viable cells of the blastocyst and an increase in these cells leads to thinning of the ZP thickness and hatching of the blastocyst (16, 44). More research is required to clarify the molecular mechanisms underlying quercetin function on development and quality of embryos in different conditions.

## Conclusion

The results suggest that quercetin may protect the embryos against actinomycin D so that increases the number of viable cells and decreases the number of apoptotic cells, which can help the expansion of the blastocysts, thinning of the ZP thickness and increasing the hatching rate in mouse embryos.

## Note

***This article extracted from M.Sc. thesis. (Sara Sadat Javadinia)***
